# Addressing Inter-Gene Heterogeneity in Maximum Likelihood Phylogenomic Analysis: Yeasts Revisited

**DOI:** 10.1371/journal.pone.0022783

**Published:** 2011-08-05

**Authors:** Jaqueline Hess, Nick Goldman

**Affiliations:** 1 Department of Organismic and Evolutionary Biology, Harvard University, Cambridge, Massachusetts, United States of America; 2 EMBL-European Bioinformatics Institute, Wellcome Trust Genome Campus, Hinxton, United Kingdom; University of Sheffield, United Kingdom

## Abstract

Phylogenomic approaches to the resolution of inter-species relationships have become well established in recent years. Often these involve concatenation of many orthologous genes found in the respective genomes followed by analysis using standard phylogenetic models. Genome-scale data promise increased resolution by minimising sampling error, yet are associated with well-known but often inappropriately addressed caveats arising through data heterogeneity and model violation. These can lead to the reconstruction of highly-supported but incorrect topologies. With the aim of obtaining a species tree for 18 species within the ascomycetous yeasts, we have investigated the use of appropriate evolutionary models to address inter-gene heterogeneities and the scalability and validity of supermatrix analysis as the phylogenetic problem becomes more difficult and the number of genes analysed approaches truly phylogenomic dimensions. We have extended a widely-known early phylogenomic study of yeasts by adding additional species to increase diversity and augmenting the number of genes under analysis. We have investigated sophisticated maximum likelihood analyses, considering not only a concatenated version of the data but also partitioned models where each gene constitutes a partition and parameters are free to vary between the different partitions (thereby accounting for variation in the evolutionary processes at different loci). We find considerable increases in likelihood using these complex models, arguing for the need for appropriate models when analyzing phylogenomic data. Using these methods, we were able to reconstruct a well-supported tree for 18 ascomycetous yeasts spanning about 250 million years of evolution.

## Introduction

Phylogenomic methods have become a standard approach to resolving species phylogenies. Classic molecular systematic methods rely on one or a few genes that are considered to be phylogenetically informative such as ribosomal RNA or mitochondrial genes. In contrast, genome-wide analysis tries to utilize the maximum amount of information encoded in multiple genomes to reconstruct inter-species relationships [1]. By combining data from different parts of the genomes we try to minimize the effect of sampling error which is encountered when a small number of characters (e.g. single genes) is analyzed and which can affect phylogenetic reconstruction.

While phylogenomic approaches surpass these stochasticity issues, they are often hampered by other sources of error. Those include between-gene heterogeneity in the evolutionary process, accuracy of multiple sequence alignments, gene- and taxon-sampling and gene duplications and losses that can all result in conflicting signal (see [2] for an in-depth review). In addition to methodological problems, processes such as independent-lineage sorting and horizontal gene transfer can lead to the most likely gene tree being incongruent with the species phylogeny, adding “biological noise” [3]. In theory, the the majority of these issues are equally problematic for single-gene phylogenetics (but see [2]). Between-gene heterogeneity however is specific to phylogenomic studies is the focus of this study.

In a classic phylogenomics study encompassing 106 genes from seven species of yeast, Rokas *et al.* [4] concluded that a supermatrix analysis, where all individual gene alignments were concatenated into a “superalignment” and analyzed using a standard evolutionary model, could give a confident species tree where individual analysis of the genes failed to find a congruent solution. It is well-known, however, that heterogeneities in the evolutionary process within single genes, such as different substitution rates across sites, can markedly affect phylogenetic reconstruction (e.g. [5], [6], [7]). So in order to gain maximum profit from the increased amount of data, which can only be expected to increase heterogeneity as data from different regions of the genomes are included, it is necessary to use appropriate models that deal with variations in the evolutionary processes across different loci and between different species.

As increasing amounts of data from yeasts and other organisms are becoming available, it is an appropriate time to consider whether supermatrix methods are still practical and reliable when applied to larger datasets like those that we are able to assemble today. We have investigated this, and generated a phylogeny that is robust to the effects of between-gene variation, by revisiting a classic problem in yeast phylogenomics.

### Considerations with the supermatrix approach

There are a number of issues that can influence the accuracy of phylogenetic reconstruction in general. The most well-studied among those is probably across-site rate variation [8] which is typically accommodated by adding gamma-distributed rates to the evolutionary model [9]. Patterns of substitutions between different residues are also known to differ depending on their physiochemical properties and placement within the protein structure. Those differences are accounted for by increasingly complicated models of nucleotide substitutions such as those used in this study and by a variety of amino acid replacement matrices (e.g. [10]–[12]) as well as mixture models (e.g. [13]). In addition to variation in space, variation of the evolutionary process in time such as site-specific rate variation across lineages, referred to as heterotachy [14] can adversely affect the outcome of phylogenetic reconstruction. Whilst some solutions towards accommodating those processes are beginning to appear (e.g. [15], [16]), they remain less well understood and are computationally expensive. Other issues that are difficult to account for *per se* include compositional bias [17] and mutational saturation that can lead to long branch attraction artifacts [18].

Seeing that the effects of those factors are already apparent even in single-gene studies, they can only be expected to gain in impact when data from multiple genomic regions are being analysed. Even when we account for processes such as different rates across sites, concatenation and subsequent supermatrix analysis using an evolutionary model with a single set of parameters for the entire dataset is assuming homogeneity, or rather a “constant heterogeneity”, of the evolutionary process.

This is highly unlikely to hold and it has been shown that systematic errors resulting from the model violations mentioned above can be exacerbated by concatenation to the extent where highly-supported but incorrect topologies are recovered [5]–[7]. A number of treatments to mitigate such effects have been proposed, e.g. increased taxon sampling to break up long branches and thereby reduce the effect of multiple substitutions [1]; the removal of fast-evolving species, genes or sites as those are suspected to be most prone to accumulate non-phylogenetic signal [7], [19], [20]; and recoding of data as purines and pyrimidines only (RY-coding) for nucleotides [21] or according to functional categories for amino acids, to reduce compositional bias [20]. Although these measures seem to work in some cases (e.g. [20]), they are treating the symptoms of the problems, not the causes. Typically they discard potentially informative parts of the data and it is unclear in what way this affects the accuracy and robustness of inferences.

We prefer to address heterogeneity issues by using more sophisticated models to fit our data, aiming to retain all useful information rather than discarding parts of the data to fit the models being used. Partitioned analysis in which parameters of the evolutionary model are estimated separately for each partition (in our case, each gene) in the dataset is a solution whose efficacy has recently been demonstrated in studies on simulated data [22] as well as empirically in a study of the branching order at the base of the mammals [7]. The term ‘supermatrix analysis’ often refers to the simple concatenation approach, in this paper we will use it to refer to all such approaches and distinguish these levels of complexity by denoting them as either “concatenated” (all partitions treated equally) or “partitioned” (subset of the sites treated differently).

Mixture models also provide means for addressing heterogeneities in the data by using different substitution matrices (e.g. [13], [23]) or different sets of branch lengths [15] for different pre-defined or learned partitions in the dataset. They are however computationally expensive for large datasets. Partitioned models, which are essentially an extreme case of mixture models, are computationally more feasible due to the ability to easily parallelize computation and are likely to scale well and we thus decided to focus on those.

### Yeast phylogenomics

The ascomycetous yeasts have been the focus of many smaller [24]–[27] and larger [4], [19], [21], [28]–[33] multigene studies. While the smaller studies cited encompass a large range of species, they incorporate only a handful of purposely sequenced genes in their supermatrix analyses and thus add relatively little extra data. The first study attempting larger scale was conducted by Rokas *et al.* [4] with 106 genes, focussing on the relationships within the *Saccharomyces sensu stricto* species only. Fitzpatrick *et al.* [29] extended this by using a slightly larger dataset (153 genes) and a wider phylogenetic range across the Ascomycota. More recent studies [30], [33] further increased the number of genes and species studied, analysing concatenated datasets of 531 and 1137 genes in 21 species respectively.

With the exception of the studies presented in [25], [28], [26] and [27] all supermatrix analyses mentioned above have been carried out in a concatenated manner. Some (e.g. [4]) do not address systematic error at all; others try to account for non-phylogenetic signal simply by removal of fast-evolving sites or by RY-recoding [19], [21], [29]. As mentioned earlier, however, those treatments are not well-suited for a comparative analysis and do not directly address heterogeneity issues between the concatenated genes. In order to examine the species tree for 18 ascomycetous yeasts in the light of such potential heterogeneities and to investigate how more data contribute towards solving more difficult phylogenetic problems, we have extended the well-known phylogenomics study of Rokas *et al.* [4] by considering 10 additional species. This increased the diversity to a range of species that shared their last common ancestor about 250 million years ago while approximately trebling the number of genes to 343. This represents a phylogenomic dataset of a scale that is more typical of the problems that are studied today. While this number is smaller than the number of genes studied in previous studies [30], [33], we have aimed to collect a dataset of high quality by omitting genes belonging to large gene families which are prone to spurious orthology assignments, especially when the annotation of some of the genomes included is of low quality [34].

Furthermore we want to examine the effects of more sophisticated models accounting for both intra- and inter-gene heterogeneity of evolutionary dynamics, especially with regards to the conclusions drawn by Rokas *et al.* [4], who claim to have obtained high-confidence results from rather simplistic analysis. It is known that over-simplification can lead to over-confidence [35], [36] and it is interesting to see if those conclusions hold for more complex datasets and analyses such as ours.

The nucleotide sequences of the 343 genes were analyzed both individually and as a supermatrix. We explored the signals present in the data when analyzed as single genes and determined the impact of model choice. We examined the validity of using a supermatrix analysis on a dataset of this scale and investigated more sophisticated maximum likelihood (ML) analyses, accounting for heterogeneities between the genes while considering the entire dataset. Furthermore we were able to achieve robust estimates of controversial regions of the phylogeny using thorough modeling of inter-gene heterogeneity in supermatrix data. Analysis of the 343 genes' amino acid sequences confirmed these results.

## Methods

### Data collection and preparation

The core of the species we selected for analysis were the eight species that were included in the Rokas *et al.* [4] study. We considered 10 additional, more divergent, ascomycetous yeasts including the well-studied pathogenic fungus *Candida albicans* and the distantly related *Yarrowia lipolytica* as an outgroup [37]. Primary analyses were performed on nucleotide coding sequences. In order to reinforce the results obtained with the nucleotide dataset in concatenated and partitioned supermatrix analyses, we repeated those using amino acid data.

We obtained orthologs from the Fungal Orthogroups Repository (FOR) at the Broad Institute [38] which contains orthology assignments of protein-coding genes for 14 out of the 18 species considered in this study. The orthology assignments available in FOR are results of computational synteny-asissted homology reconstruction [38] but also incorporate curated homology assignments from the yeast gene order browser YGOB [39] and are therefore more robust to erroneous grouping of paralogs due to reciprocal gene loss. We screened FOR for groups of orthologous genes (“orthogroups” hereafter) with exactly one representative in each of the species studied that were included in FOR, resulting in an initial list of 1148 orthogroups.

For the purpose of mapping those orthogroups to the remaining four species that were not included in FOR we used the amino acid sequence of the representative *S. cerevisiae* protein for each orthogroup to search against the remaining four genome sequences using tblastn [40]. In order for an orthogroup to be considered for further analysis we required it to be complete, i.e. containing all 18 species, as well as sufficiently divergent in order to avoid the possibility of unknowingly analyzing paralogous sequences and thereby introducing bias. The last point was addressed by further tblastn searches against the respective protein annotations using the *S. cerevisiae* member of each orthogroup. If each of the respective orthogroup members was found to be the best hit in its genome and its blast score was at least twice that of the next match the orthogroup was considered sufficiently divergent.This filtering step led to a reduction in the number of orthogroups to 629.

Within each orthogroup, the amino acid sequences were mapped to the corresponding genomes and their nucleotide sequences were extracted if we could find an exact match to the respective genome sequence. We again filtered the orthogroups by requiring nucleotide sequences for all 18 species to be present, further reducing the set of orthogroups to 357. This represents a large reduction in the number of orthogroups and was mainly due to the *Candida albicans* genome release used in this study (Assembly 20) which we later discovered was a superposed “mosaic” haploid assembly of the previous diploid assembly (Assembly 19). A final six orthogroups were removed due to convergence problems in phylogenetic analyses (see below).

Upon release of the current version of FOR (release 1.1), which included updated annotations for *Saccharomyces kluyveri* and *Lodderomyces elongisporus*, we reexamined the orthogroups we had collected for analysis. Overall, we found changes in orthology assignment in 14 orthogroups which were updated accordingly, and a further seven orthogroups that were no longer in agreement with the conditions outlined above were removed from our analysis.

The amino acid orthogroups were aligned using Mafft version 6.24 [41]. Regions of potentially low quality in the alignments were removed using Gblocks version 0.91b [42], using default parameters apart from the minimum number of sequences for a flank position which was set to 10; the minimum block length, set to 5; and gaps were allowed for half the sequences. Arguably, trimming alignments in this manner may also discard potentially useful information but we considered this necessary to avoid introducing conflicting signal through misalignment. We used BLAT [43] to map the trimmed alignments to their respective genomic location, to create the nucleotide alignments of the coding sequences used in all further analyses.

We consider these data collection and filtering procedures to be stringent, and used them in order to avoid introducing any confounding sources of error whilst trying to be as inclusive as possible. The trimmed nucleotide and amino acid alignments used in this study are available on request.

### Evolutionary Models

**Figure 1:**
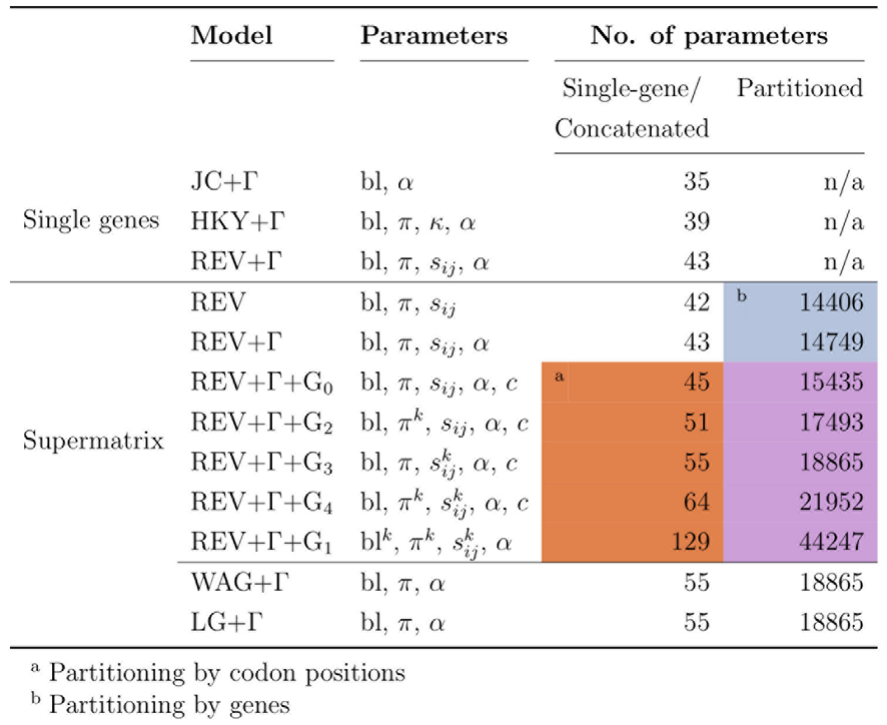
Evolutionary models used and the number of parameters estimated per model. The number of parameters for partitioned analyses is based on the dataset size of 351 genes, where each parameter is estimated separately for each gene. 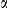 is the shape parameter of the gamma distribution, bl are the branch lengths, 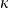 and 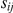 are the rate ratio parameters (see Methods) and 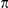 are the nucleotide frequencies. When the Mgene options in baseml are used, 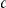 represents two scaling factors for proportional branch lengths at different codon positions and the “
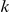
” superscript indicates that in these models, those parameters are estimated separately for each codon position. The area shaded in red (note a) indicates partitioning by codon positions, blue (note b) indicates partitioning by genes and purple indicates partitioning by both codon positions and genes.

We tested an array of combinations of evolutionary models (with varying degrees of complexity) and partitionings of our data, summarized in Figure 1. The Jukes-Cantor model (JC; [44]), being the simplest of all those evolutionary models, assumes equal nucleotide frequencies and no difference in rate between transitions and transversions. The Hasegawa-Kishino-Yano model (HKY; [45]) parameterizes the different nucleotide frequencies (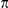) and includes a rate ratio parameter (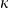) for the ratio of the rates of transition and transversion substitutions. Finally, the general time reversible model (REV; [46], [47]) includes parameters for the different nucleotide frequencies (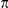), as for HKY, as well as exchangeability parameters (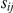; [48]) for every possible type of substitution (also referred to as the exchangeability parameters 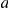
–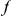 in the PAML package [49]). Among-site heterogeneity in the rate of evolution was modeled using a discrete gamma distribution (+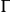 with parameter 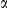; [9]) and six rate categories.

Supermatrix analyses were performed using a range of nested models, each increase in complexity allowing for more amongst-genes heterogeneity. Here, REV was chosen as the basic model of evolution (see below) and we successively added a discrete gamma distribution and separate categories for the different codon positions to allow for the differences in the evolutionary process experienced between the first, second and third codon position.

Partitioning of codon positions (see Note a in Figure 1) was carried out using the “Mgene” options implemented in baseml from the PAML package [50]. G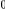 is the simplest of those options, introducing parameters for different rates at each codon position in the form of branch length scaling factors (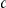). G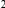 and G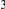 additionally estimate separate nucleotide frequencies or separate exchangeability parameters for each codon position, respectively (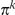 or 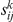 for 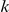 = 1, 2, 3); G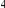 estimates separate nucleotide frequencies, exchangeability parameters and different rates (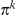, 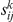, 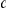). Finally G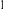, the most general option, estimates separate nucleotide frequencies, rate ratio parameters and different non-proportional branch lengths (bl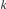) for each position.

To address between-gene heterogeneities, partitioning by genes was performed with both unpartitioned genes (rows 1 and 2 in note b; Figure 1), and in addition to partitioning by codon positions (rows 3 to 7 in note b; Figure 1). Due to the capabilities of available software and the computational cost involved, we could only carry out the partitioning between genes by calculating likelihoods individually for each gene and then summing over the trees tested, treating genes entirely independently: this equates to Mgene option G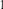. The most complicated model thus estimates separate branch lengths, nucleotide frequencies and exchangeability parameters for each codon position in every gene as well as a separate 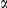 for each gene in the dataset.

Amino acid analyses were carried out using he WAG and LG models of evolution [10], [11] with a gamma distribution (again using six rate categories).

### Single-gene analyses

We used ML phylogenetic methods for all of our analyses due to their power and ability to explicitly model different evolutionary patterns both within and between genes [51]. Gene trees were built using Leaphy 1.0 [52]. We estimated trees using the JC, HKY and REV nucleotide models of evolution (Figure 1, “Single genes” rows). Rate heterogeneity among sequence positions was modeled using a gamma distribution [9]. In order to assess confidence in the individual nodes of the tree we performed non-parametric bootstrap analyses with 100 replicates each [53].

### Supermatrix analysis

We chose baseml [50] to perform the supermatrix analyses of our dataset due to the wealth of models it implements. As baseml lacks sophisticated tree search algorithms, we specified a candidate tree set for exhaustive analysis (CTS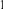; Supplementary Data S1) based on prior knowledge about the evolutionary relationships in some parts of the phylogeny (see below).

**Figure 2:**
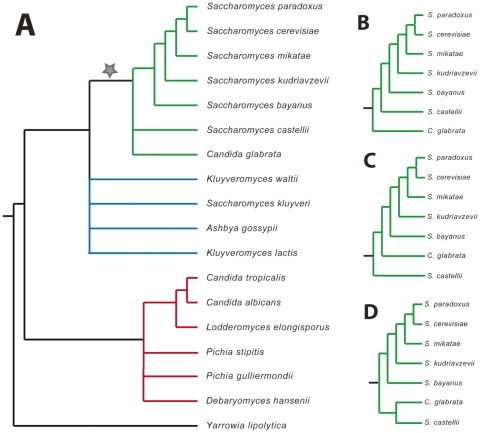
Species considered in this study and their phylogenetic relationships. **A:** “Backbone” species tree based on well-accepted phylogenetic relationships and secondary genomic features (see Methods). Branches colored in green indicate the post-WGD clade, blue the pre-WGD clade and red the CTG clade. **B**–**D:** Alternative resolutions recovered for the post-WGD clade.

Figure 2A shows the “backbone” species tree of the 18 yeasts we studied. The three major clades shown in different colors in Figure 2A are stably recovered by sequence analysis and are further supported by other shared genomic features, namely the 2∶1 syntenic correspondence of the “post-WGD” species (green) to the “pre-WGD” species (blue) [39] and a change in the genetic code, translating the CTG codon into serine instead of leucine on the lineage leading to the *Candida* clade (red; [54], [55]). These well-accepted relationships are shown fully resolved, whereas regions of uncertainty in the phylogeny are collapsed into polytomies. CTS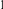 initially included all trees found by resolving all the polytomies shown in the tree into all possible arrangements. In order to keep the number of topologies manageable, we initially resolved the relationship between *Candida glabrata* and *Saccharomyces castellii* as shown in Figures 2B and 2C, resulting in 3150 topologies. We then ran an initial analysis using the partitioned REV+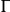
+G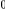 model (see Figure 1) that was found to be a good model for supermatrix analysis of nucleotide data (as described below). In order to test whether the possible resolution shown in Fig. 2D need be further considered, we identified the 500 best-scoring topologies from these analyses, modified those to incorporate the resolution shown in Fig. 2D and performed further ML analysis using the same model. Based on this analysis, we excluded the possibility of *C. glabrata* and *S. castellii* branching as sister species (Figure 2D) due to consistently very low likelihoods for those trees (results not shown).

Both nucleotide and amino acid analyses were carried out on a concatenated as well as a partitioned form of the data using the CTS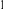 and the evolutionary models described in Figure 1 (“Supermatrix” rows). Bootstrap analyses were performed using the RELL method [56] with 1000 samples for all of the above analyses.

### Model and tree comparison

The likelihood ratio test (LRT, [57]), a widely-used statistic for model selection, was used to perform hypothesis testing between two nested models. In addition to LRTs we also calculated the AIC scores [58], allowing for the comparison of non-nested models [59]. Because our sample size was small in comparison to the number of free parameters (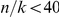) we used 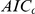, the second order approximation of the AIC score, for our tests [60]. As additional parameters for partitioned models are added for each partition rather than the entire dataset, we calculate the penalty term on a per-partition basis:
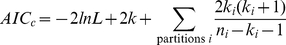
(1)where 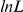 is the maximized log-likelihood of the data, 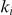 is the number of parameters estimated for partition 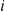 with 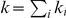, and 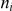 is the sample size (number of alignment positions) for partition 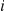 with 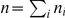.

The AIC score is known to favor parameter-rich models under some conditions while the BIC score [61] is generally considered to be more conservative [60], [62]. In order to obtain a conservative estimate of model-fit we repeated model testing using the BIC as detailed in the Supplementary Text S2.

In order to gauge the heterogeneity inherent in our dataset, we examined the distributions of estimates of two parameters included in our models and the average GC content across alignments. These estimates were taken from our single-gene analyses under the REV+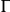 model of evolution, which was found to be optimal in single-gene analyses (see below). The average transition/transversion ratio *R* [51] for a gene is given by:
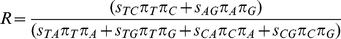
(2)where 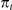 are the nucleotide frequencies and 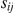 are the exchangeability parameters as defined by the REV model [48].

Considering the distributions of *R* and 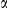, the shape parameter of the gamma distribution [9], over all genes presents a way to display the diversity of the parameterization of the same model for different genes and hence heterogeneity between them. Similarly, the differences in GC content between genes reflect an aspect of between-gene heterogeneity that is ignored when the supermatrix is analyzed using a single parameterization.

We measured the difference between estimated trees using the normalized version of the Robinson-Foulds (RF) distance [63].

## Results and Discussion

### Single-gene analyses

#### The influence of model choice on gene tree reconstruction

To investigate the impact of model choice on tree reconstruction we analyzed the 343 gene nucleotide dataset using the JC + 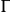, HKY + 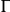 and REV + 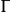 models of evolution. We present only results for models including gamma-distributed intra-gene rate heterogeneity since these models were always significantly preferred (results not shown). When we examined how often the ML trees for a gene using the different models were identical, we found the topologies recovered using the HKY + 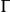 and REV + 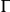 models to be the same for about 47% of the genes. In contrast, the number of identical topologies recovered when REV + 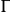 results were compared to JC + 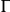 results was much smaller, with the same ML tree obtained for just 8% of genes. Similarly, JC + 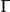 and HKY + 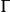 analyses resulted in 9% of shared trees. This discrepancy was also detected when we investigated the degree of differences between the trees recovered by the different models for a given gene. On average, the normalized RF distance (RF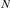) between the REV + 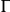 and the HKY + 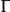 topologies was 0.08 whereas RF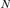 between REV + 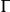 and JC topologies was 0.27.

Examination of the bootstrap support for conflicting nodes in alternative trees recovered by different models showed few strongly supported conflicts when comparing the REV + 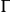 and HKY + 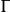 topologies (see Supplementary Figure S1C), in line with recent results by [64] who found the differences between trees constructed with alternative well-fitting models to be of little importance. The support for conflicting nodes between REV + 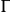 and JC + 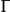 trees is markedly higher (Suppl. Fig. S1B). The increase in congruence and the decrease in well-supported conflict when more complex models are used both show that choosing a better model improves results and that it is important to choose a model that best fits the data.

#### Best-fit models

We used hierarchical LRTs to determine the optimal model for each gene. HKY+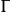 was always found to be better than JC+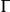 and REV+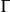 was found to be the best-fitting model for all of the 343 genes studied. We consequently focussed on REV+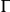 as the model for analysis of our nucleotide data. Nevertheless, even with the best available models there remain discrepancies that can affect downstream analyses, as is shown next.

#### Large amounts of incongruence among the single-gene datasets

We now concentrate on the results of analyzing the single-gene datasets using the optimal model found for each gene (see above). We found very large amounts of incongruence within the set of ML trees recovered. In total we obtained 336 distinct ML topologies for the 343 genes: in other words, a different phylogeny of the 18 yeasts for almost every individual gene. The mean pairwise RF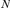 distance among the 336 topologies is 0.54. So whilst they are clearly more similar to each other than 336 randomly drawn trees of the same size (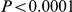 from 10000 simulations; mean RF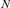 distance 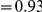, std. dev. 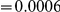), it still means that on average the gene trees for any two genes differ by 16 unique bipartitions.

The analysis of single genes proved inconclusive in our case and we were unable to derive a species phylogeny supported by the individual gene phylogenies. Incongruence among gene trees is not specific to our dataset but instead is found in a large fraction of studies comparing single-gene phylogenies [65]. Even though it is as yet unclear how much incongruence between gene trees in a genome is “normal”, the level of incongruence we have encountered (i.e. 336 phylogenies from 343 genes) is surprising. In order to confidently resolve inter-species relationships we need a way of combining the data in a sensible manner. Supermatrix analysis promises resolution of conflict where individual analyses fail.

**Figure 3:**
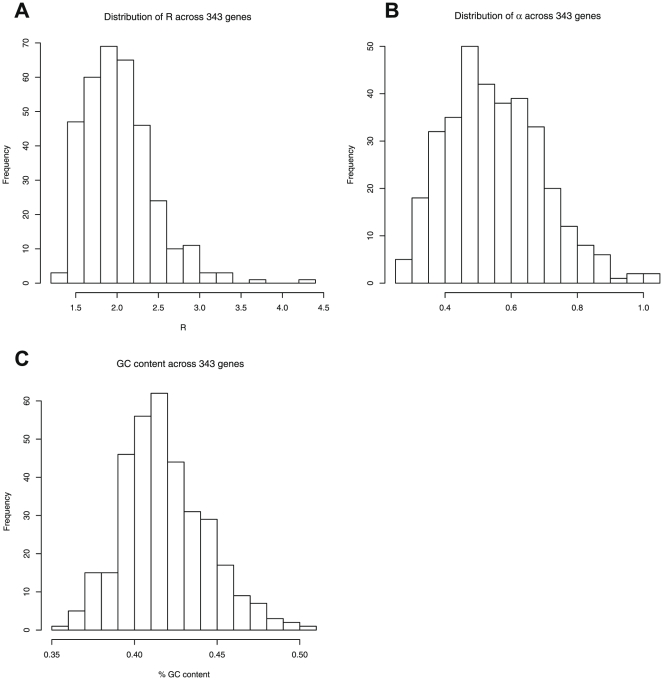
Heterogeneity of parameters estimated across single genes. **A:** ts/tv ratio (*R*) and **B:** gamma-distribution shape parameter 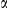, estimated on the ML topology for each of 343 genes (see Methods).

To illustrate the heterogeneity across the single-gene datasets and thereby speculate about the validity of a concatenation approach for further analysis we investigated distribution of the transition/transversion ratio *R* (Fig. 3A), 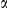, the shape parameter of the 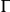 distribution used to model among-gene rate variation (Fig. 3B) and the average GC content (Fig. 3C) for each of the genes. Each of these measures demonstrates a potential source of inter-gene variation in evolutionary dynamics that we were able to account for in supermatrix analysis using partitioned models (see Figure 1). We used the parameter estimates of each gene under the REV+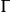 model of evolution as calculated by baseml.

The lower and upper 95% percentiles of *R* are 1.48 and 2.81 respectively while the full range of *R* is large, extending from 1.30 to 4.22. This is similar to previously compiled distributions of *R* over a range of different genes [66]. Similarly, 95% of the estimates of 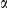 fall between 0.34 to 0.81 while again the full range extends from 0.25 to 1.03, showing that variation between genes in rate heterogeneity across sites can also be considerable. GC content follows a similar trend, with lower and upper 95 percentiles at 38% and 47% average GC content respectively. The level of variation for all three of the examined parameters, although not extreme, is considerable and it is currently unclear how much of between-gene heterogeneity is sufficient to result in model violation when genes are concatenated.

In order to assess whether extreme values of the distributions of *R* and 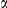 are simply the result of noisy parameter estimation we investigated their distribution with respect to alignment length and standard errors. Although there is a weak association, as would be expected, it does not appear to account for the extreme ranges of these distributions (see Supplementary Text S1; Supplementary Figure S2).

The number of distinct topologies recovered in single-gene analyses underlines the need for phylogenomic methods to distill the shared “historical” signal between the genes analyzed. At the same time, the level of heterogeneity encountered between the genes when examining three key parameters of the optimal evolutionary model suggests that a simple concatenation supermatrix approach might not be valid and complex models are needed to analyze a dataset containing this much variation.

### Supermatrix analysis

#### Complex data require complex models

We performed supermatrix analyses using a range of different models and partitions of increasing complexity, allowing for amongst-gene and between-gene heterogeneity (Fig. 1, Table 1). The optimal model was determined using LRTs where applicable and 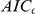 as well. The order in which LRTs for the different Mgene options were performed is as follows; G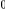 - (G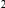,G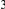) - G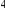 - G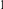. G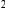 and G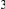 are not nested and can thus not be tested against each other in a LRT. The results of this are shown in Table 1. The most comprehensive model, partitioned REV+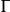
+G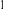, was found to be optimal for our supermatrix – reflecting the complexity of the signal encoded in these data. (Comparisons using the more conservative BIC differ very slightly– see Supplementary Text S2; Supplementary Figure S3.)

**Table 1:** ML trees and test statistics for model tests performed on the supermatrix dataset.

	Concatenated			Partitioned		
Model	ML tree	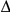 AIC	P(LRT)	ML tree	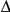 AIC	P(LRT)
REV	A	990359	-	A	872007	-(0*)
REV+ 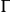	B	431437	0*	B	315551	0*(0*)
REV+ 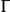 +G 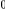	C	283288	0*	C	155540	0*(0*)
REV+ 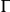 +G 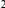	C	279792	0*	C	146131	0*(0*)
REV+ 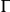 +G 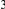	C	162718	0*	C	30691	0*(0*)
REV+ 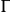 +G 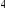	C	144273	0*/0*	C	7156	0*/0*(0*)
REV+ 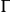 +G 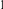	C	123756	0*	C	0	0*(0*)
WAG+ 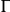	C	74186	-	C	25394	-(0*)
LG+ 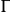	C	52235	-	C	0	-(0*)

ML trees and test statistics for model tests performed on the supermatrix dataset. Models used are as in Figure 1. LRTs were performed between the model considered and the next-smallest nested model. Models with Mgene option G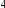 were tested against both G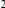 and G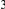 models. 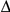
AIC is the difference in 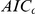 between a model and the best-fitting model. Partitioned models were also tested against their concatenated version (in brackets). Significant P-values (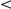
0.001) are indicated by a star. Trees A and B are shown in Supplementary Figure S4.

Model testing and the choice of an appropriate model proved to be important in our case, because the ML tree again changes depending on which model is used (Table 1). Both different rates across sites (+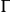) as well as different rates at each codon position (+G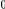) influenced which tree was found to be the ML tree. Interestingly, as more parameters are free to vary at different codon positions the likelihood increases but the ML topology remains the same, reinforcing our confidence in having found a good species tree (see Table 1).

It is also noteworthy that the bootstrap support across the ML trees recovered using the overly-simple REV and REV+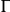 models (Suppl. Fig. S4, trees A and B) is high and thus it is not acting as a reliable indicator when trying to assess the confidence for trees recovered in our analyses. In general, large amounts of data can give over-confidence in the result obtained when there is model mis-specification and, in particular, in cases of over-simple models [35]. Great care should be taken to find the best available model for any given dataset.

#### Partitioned analysis outperforms concatenated analysis

All models we tested were implemented both using a conventional concatenation approach where a single parameterization of the evolutionary model is used to analyze the entire dataset, as well as more sophisticated partitioned analysis where parameters are estimated for each partition (in this case genes), thus allowing for more amongst-genes heterogeneity.

**Figure 4:**
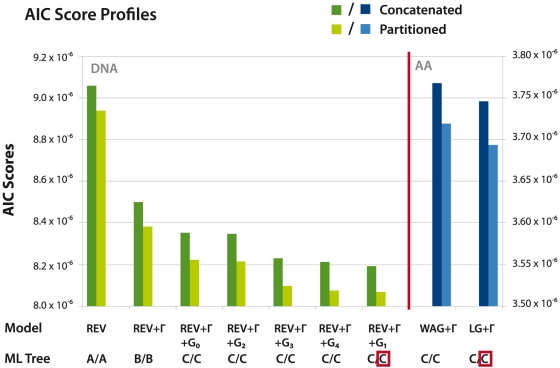
AIC score profiles for supermatrix analyses, differentiated by evolutionary model and type of analysis. Partitioned analysis (light colors) consistently outperformed concatenated analysis (dark colors). The choice of a partitioned vs. concatenated model does not affect which tree was found to be optimal when analyzing nucleotide data (green) as well as amino acids (blue). The ML topology obtained using the optimal model for both nucleotide and amino acid data is the same (red boxes) and is depicted in Figure 5. Trees A, and B are depicted in Supplementary Figure S4.

Partitioned analysis consistently outperformed concatenated analysis independently of the model of evolution used (Table 1; Figure 4). LRTs were highly significant and the difference in 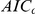 between partitioned and concatenated analyses is substantial for all models, representing a major improvement in model fit. In contrast to results obtained with a mammalian dataset [7], the use of partitioned versus concatenated analysis had no effect on which topology was found to be optimal in the nucleotide analyses – a poor choice of model (e.g. REV or REV+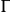) still leads to a sub-optimal tree, even if partitioned analysis is used. Model testing using BIC confirmed those results (see Supplementary Information).

#### Amino acid analyses

**Figure 5:**
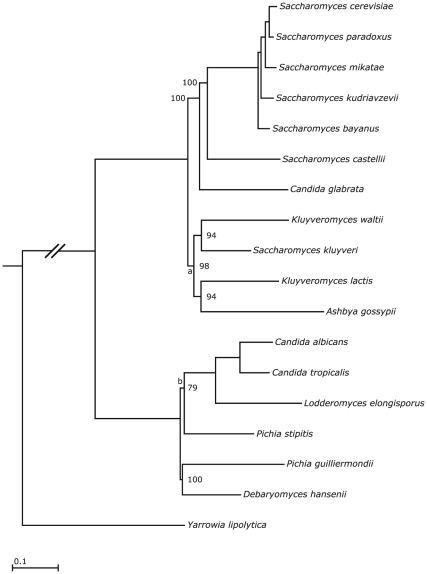
ML tree obtained using the optimal nucleotide and amino acid models of evolution (tree C in **Fig. 4**). Bootstrap values are from 1000 iterations of RELL resampling and branch lengths, in expected number of substitutions per nucleotide, are calculated as the weighted mean of individual estimates in partitioned analysis of the 343 genes of the nucleotide datasets. The branches marked by lowercase letters were extended for the purpose of visualization.

Amino acid-level analysis are less prone to mutational saturation and the effects of base composition variation between different genome sequences that may affect nucleotide data [19]. Although appropriate nucleotide-level models should be able to account for such effects, in addition to analyzing the nucleotide dataset we also investigated supermatrix analyses on the translated amino acid sequences. We used the WAG+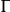 model [10] as well as LG+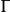 [11] to perform concatenated and partitioned analyses of CTS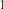 using the codeml program from the PAML package [50]. The LG+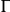 model was preferred by LRT and the 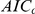 criterion. Again we found the partitioned model to outperform the concatenated model, with significant improvements in likelihood confirmed by both LRT and 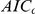 (Table 1; Figure 4). (The alternative BIC criterion disagrees in this one case, see Supplementary Text S2, but this does not affect the proposed ML topology.) The proposed ML topology was found to be identical across all partitioned and concatenated amino acid analyses and identical to the one obtained using the best nucleotide model (Fig. 5), reinforcing the results obtained with the nucleotide dataset.

#### Species phylogeny of 18 ascomycetous yeasts

The species phylogeny we obtained in our supermatrix analysis is shown in Figure 5. It receives high bootstrap support across the tree. The pre-WGD species were recovered as a monophyletic clade where *Saccharomyces kluyveri* and *Kluyveromyces waltii*, and *Ashbya gossypii* and *Kluyveromyces lactis*, respectively, are sister species that form sister clades to one other. This agrees with some of the more recent large phylogenomic studies [19], [29] but is in contrast to results obtained by smaller studies (e.g. [24], [31]) which both infer *A. gossypii* at the base of the remaining pre-WGD species. We infer *Pichia stipitis* to be basal to the *Candida* species and *Lodderomyces elongisporus* although bootstrap support for this node is relatively lower and the branch length very short, retaining the possibility of alternative placement at the base of the clade containing the *Debaryomyces hansenii* and *Pichia guilliermondii*.

The branching order at the base of the WGD clade in our ML tree sees *C. glabrata* splitting off before *S. castellii*. This is in disagreement with the branching order inferred from synteny data [67] but also frequently recovered by other supermatrix-based phylogenomic analyses of this clade, especially when a large number of genes was analyzed (e.g. [19], [30]–[33]). As above, this stands in contrast to the smaller multigene analyses (e.g. [24], [27]). When we examined the support for either of three possible branching orders (*C. glabrata* first and *S. castellii* second or *vice versa*, or with *S. castellii* and *C. glabrata* as sister species) in our single-gene dataset (REV + 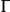) we found the first branching order to be most strongly represented amongst the genes trees (150 occurrences) in relation to the other two branchings (66 and 58 occurrences, respectively). The study of substitution patterns hence consistently suggests *C. glabrata* as the species at the base of the WGD clade.

This disagreement of substitution patterns with synteny information could be due to the fact that, as yet, we have limited knowledge of how to statistically and reliably estimate phylogenies from chromosome rearrangement data. Alternatively, it is possible that the observed discrepancies are the result of biological processes such as independent lineage sorting (reviewed in [3]) or introgression (e.g. [68]), especially seeing that the divergence time between *S. castellii* and *C. glabrata* is relatively short [67].

### Conclusions

The power of phylogenetic reconstruction is heavily dependent on the evolutionary models being utilized. This is already well-known (e.g. [35], [36], [64]) and it is thus not surprising to find model choice having a large impact in phylogenomics.

Our datasets deliver another example where single-gene phylogenetics fails to find a congruent solution when trying to resolve the species tree that underlies the evolution of those genes. In fact, in this difficult phylogenetic problem the number of proposed ML trees increases almost linearly with the number of genes studied. Phylogenomics approaches are well-suited to address such incongruencies and we would like to see them deliver resolution even as datasets increase in size and diversity. Their ability to do so has been demonstrated in the past, largely on smaller and easier studies (e.g. [4], [24], [25]). Given our results, it appears that this remains true for a dataset of our size and complexity, but with the qualification that it is vital that analyses appropriate to the complexity of the data are used.

We have examined the impact of inter-gene heterogeneity in phylogenomic analysis of yeasts by using partitioned models of evolution, accounting for between-gene variation in the parameters modeled. To our surprise, we found that the modeling of differences between genes did not influence the identity of the ML topology compared to a concatenated analysis using the same model of evolution. Nevertheless, there were considerable improvements in likelihood when the data were partitioned on a gene by gene basis, confirming the significance of inter-gene variation of evolutionary dynamics.

While the identity of the ML topology was not affected by the choice of a partitioned over a concatenated model, it was highly dependent on the evolutionary model employed, resulting in a different ML tree for all three types of model (no heterogeneity; among-site heterogeneity; and among-site heterogeneity plus individual treatment of codon positions) used. This indicates that the heterogeneity of the evolutionary process affecting substitution patterns within a single protein-coding gene resulted in stronger (misinterpreted phylogenetic) signal than the differences between such processes acting on different loci across the genomes of 18 yeast species.

This also underlines the effectiveness of model-based approaches in addressing heterogeneous phylogenetic signal as is demonstrated by the fact that we are able to recover the same phylogeny for the nucleotide data as in the amino acid analysis, once the differences in evolutionary rates between codon positions are accounted for.

We obtained a fully-resolved species tree for 18 ascomycetous yeasts that receives high bootstrap support and is robust to between-gene variation of the evolutionary processes accounted for here. We were able to confirm the relationships in the pre-WGD species as a monophyletic clade as well as resolve the branching order within the clade containing the *Candida* and *Pichia* species. Our analysis suggests *C. glabrata* to be at the base of the WGD clade.

The topologies we recover for the pre-WGD species and the branching order at the base of the WGD are commonly recovered in larger phylogenomic analyses (e.g. [19], [29], [33]) but sometimes disagree with those recovered from smaller analyses (e.g. [24], [27]). In the case of the pre-WGD species this may be a taxon sampling issue, seeing that smaller studies often consider more species (e.g. [24]) that might help to resolve the deep divergence between the pre-WGD species (see Fig. 5).

The discrepancies about the branching order at the base of the WGD species, however, remain more difficult to explain. Both our single-gene and supermatrix analyses provide overwhelming support for *C. glabrata* to be at the base of the WGD species. Nevertheless, we find support for either of the alternative resolutions (Fig. 2B and C) in approximately a quarter of the single-gene trees respectively, suggesting the presence of non-tree like inheritance through e.g. introgression [68] or the strong influence of population genetic processes.

Methods that take into account population genetic processes that can lead to incongruence between gene trees and the species tree have recently become available (reviewed in [3]). Those are also still computationally expensive for phylogenomic analysis but might help the resolution of shallow divergences, such as the branching order of *C. glabrata* and *S. castellii*, in the future.

It is known that sources other than inter-gene heterogeneity can affect the accuracy of phylogenetic reconstruction. These include compositional heterogeneity (e.g. [69]), heterogeneity in the pattern of substitution at different sites on the amino acid level (e.g. [13]), heterotachy [14] and mutational saturation (e.g. [19]). Models that address some of those issues (e.g. CAT: [13]) are available, but phylogenetic inference with them is computationally expensive and as such currently not feasible in an analysis of the size attempted here. It will however be interesting and necessary to assess the robustness of the species tree recovered here against other types of heterogeneous process.

Overall we are confident that phylogenomics methods, given the right evolutionary models, can give robust answers to a number of yet-to-be-resolved branches of the Tree of Life.

## Supporting Information

Figure S1 **Bootstrap support for shared and variable nodes of gene trees estimated using different models of evolution.** Gene trees for each alignment were estimated using Leaphy 1.0 with 100 bootstrap replicates each. The distribution of bootstrap values for nodes shared between the gene trees estimated using respective models of evolution are shown in blue, bootstrap values of variable nodes are shown in red.
(EPS)

Figure S2
**Distribution of the distance of** 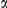 **(A) and *R* (B) to the mean of the respective distributions by alignment length.** For 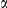, the distribution of the standard error of the estimate with respect to the alignment length is also shown.(EPS)

Figure S3 **BIC score profiles.** BIC scores were calculated as outlined in Supplementary Text S2. As for the AIC results (Fig. 4), partitioned analysis (light colors) consistently outperformed concatenated analysis (dark colors) on the nucleotide data. Here, for amino acid data we found concatenated models to be preferred to partitioned ones however.
(EPS)

Figure S4 **Supermatrix ML trees recovered using non-optimal evolutionary models.** Lettering corresponds to the labels used in Table 1 and Figure 4 in the main text. Bootstrap values are indicated as a percentage out of 1000 replicates using RELL resampling (see Methods in main text).
(EPS)

Text S1 Additional analyses investigating the effects of model choice and between-gene heterogeneity in the single-gene dataset.
(PDF)

Text S2 BIC model testing.
(PDF)

Data S1Candidate tree set (CTS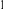) compiled for exhaustive ML analysis using PAML [50].(TXT)

## References

[1] Philippe H, Delsuc F, Brinkmann H, Lartillot N (2005). Phylogenomics.. Annual Review of Ecology, Evolution, and Systematics.

[2] Philippe H, Brinkmann H, Lavrov DV, Littlewood DTJ, Manuel M (2011). Resolving difficult phylogenetic questions: why more sequences are not enough.. PLoS Biol.

[3] Degnan JH, Rosenberg NA (2009). Gene tree discordance, phylogenetic inference and the multispecies coalescent.. Trends Ecol Evol.

[4] Rokas A, Williams BL, King N, Carroll SB (2003). Genome-scale approaches to resolving incongruence in molecular phylogenies.. Nature.

[5] Delsuc F, Phillips MJ, Penny D (2003). Comment on “hexapod origins: monophyletic or paraphyletic?”.. Science.

[6] Brinkmann H, van der Giezen M, Zhou Y, de Raucourt GP, Philippe H (2005). An empirical assessment of long-branch attraction artefacts in deep eukaryotic phylogenomics.. Syst Biol.

[7] Nishihara H, Okada N, Hasegawa M (2007). Rooting the eutherian tree: the power and pitfalls of phylogenomics.. Genome Biol.

[8] Uzzell T, Corbin KW (1971). Fitting discrete probability distributions to evolutionary events.. Science.

[9] Yang Z (1994). Maximum likelihood phylogenetic estimation from dna sequences with variable rates over sites: approximate methods.. J Mol Evol.

[10] Whelan S, Goldman N (2001). A general empirical model of protein evolution derived from multiple protein families using a maximum-likelihood approach.. Mol Biol Evol.

[11] Le SQ, Gascuel O (2008). An improved general amino acid replacement matrix.. Mol Biol Evol.

[12] Le SQ, Gascuel O (2010). Accounting for solvent accessibility and secondary structure in protein phylogenetics is clearly beneficial.. Syst Biol.

[13] Lartillot N, Philippe H (2004). A bayesian mixture model for across-site heterogeneities in the amino-acid replacement process.. Mol Biol Evol.

[14] Lopez P, Casane D, Philippe H (2002). Heterotachy, an important process of protein evolution.. Mol Biol Evol.

[15] Pagel M, Meade A (2008). Modelling heterotachy in phylogenetic inference by reversible-jump markov chain monte carlo.. Philos Trans R Soc Lond B Biol Sci.

[16] Whelan S (2008). Spatial and temporal heterogeneity in nucleotide sequence evolution.. Mol Biol Evol.

[17] Lockhart P, Steel M, Hendy M, Penny D (1994). Recovering evolutionary trees under a more realistic model of sequence.. Mol Biol Evol.

[18] Ho SY, Jermiin L (2004). Tracing the decay of the historical signal in biological sequence data.. Syst Biol.

[19] Jeffroy O, Brinkmann H, Delsuc F, Philippe H (2006). Phylogenomics: the beginning of incongruence?. Trends Genet.

[20] Rodríguez-Ezpeleta N, Brinkmann H, Roure B, Lartillot N, Lang BF (2007). Detecting and overcoming systematic errors in genome-scale phylogenies.. Syst Biol.

[21] Phillips MJ, Delsuc F, Penny D (2004). Genome-scale phylogeny and the detection of systematic biases.. Mol Biol Evol.

[22] Ren F, Tanaka H, Yang Z (2008). A likelihood look at the supermatrix-supertree controversy.. Gene.

[23] Le SQ, Lartillot N, Gascuel O (2008). Phylogenetic mixture models for proteins.. Philos Trans R Soc Lond B Biol Sci.

[24] Kurtzman CP, Robnett CJ (2003). Phylogenetic relationships among yeasts of the ‘saccharomyces complex’ determined from multigene sequence analyses.. FEMS Yeast Res.

[25] Diezmann S, Cox CJ, Schönian G, Vilgalys RJ, Mitchell TG (2004). Phylogeny and evolution of medical species of candida and related taxa: a multigenic analysis.. J Clin Microbiol.

[26] Tsui CK, Daniel HM, Robert V, Meyer W (2008). Re-examining the phylogeny of clinically relevant candida species and allied genera based on multigene analyses.. FEMS Yeast Res.

[27] Schoch C, Sung GH, Lopez-Giraldez F, Townsend J, Miadlikowska J (2009). The ascomycota tree of life: A phylum-wide phylogeny clarifies the origin and evolution of fundamental reproductive and ecological traits.. Systematic Biology.

[28] Bofkin L (2005). The Causes and Consequences of Variation in Evolutionary Processes Acting on DNA Sequences..

[29] Fitzpatrick DA, Logue ME, Stajich JE, Butler G (2006). A fungal phylogeny based on 42 complete genomes derived from supertree and combined gene analysis.. BMC Evol Biol.

[30] Kuramae EE, Robert V, Snel B, Weiss M, Boekhout T (2006). Phylogenomics reveal a robust fungal tree of life.. FEMS Yeast Res.

[31] Cornell MJ, Alam I, Soanes DM, Wong HM, Hedeler C (2007). Comparative genome analysis across a kingdom of eukaryotic organisms: specialization and diversification in the fungi.. Genome Res.

[32] Kuramae EE, Robert V, Echavarri-Erasun C, Boekhout T (2007). Cophenetic correlation analysis as a strategy to select phylogenetically informative proteins: an example from the fungal kingdom.. BMC Evol Biol.

[33] Marcet-Houben M, Gabaldón T (2009). The tree versus the forest: the fungal tree of life and the topological diversity within the yeast phylome.. PLoS One.

[34] Hess J (2011). Evolution of Transcription Factor Repertoires in the Saccharomycotina..

[35] Yang Z, Goldman N, Friday A (1994). Comparison of models for nucleotide substitution used in maximum-likelihood phylogenetic estimation.. Mol Biol Evol.

[36] Sullivan J, Swofford DL (1997). Are guinea pigs rodents? the importance of adequate models in molecular phylogenetics.. Journal of Mammalian Evolution.

[37] Dujon B, Sherman D, Fischer G (2004). Genome evolution in yeasts.. Nature.

[38] Wapinski I, Pfeffer A, Friedman N, Regev A (2007). Automatic genome-wide reconstruction of phylogenetic gene trees.. Bioinformatics.

[39] Byrne KP, Wolfe KH (2006). Visualizing syntenic relationships among the hemiascomycetes with the yeast gene order browser.. Nucleic Acids Res.

[40] Altschul SF, Madden TL, Schäffer AA, Zhang J, Zhang Z (1997). Gapped blast and psi-blast: a new generation of protein database search programs.. Nucleic Acids Res.

[41] Katoh K, Toh H (2008). Recent developments in the MAFFT multiple sequence alignment program.. Briefings in bioinformatics.

[42] Castresana J (2000). Selection of conserved blocks from multiple alignments for their use in phylogenetic analysis.. Mol Biol Evol.

[43] Kent WJ (2002). Blat–the blast-like alignment tool.. Genome Res.

[44] Jukes T, Cantor C (1969). Mammalian protein metabolism.

[45] Hasegawa M, Kishino H, Yano T (1985). Dating of the human-ape splitting by a molecular clock of mitochondrial dna.. J Mol Evol.

[46] Tavaré S (1986). Some probabilistic and statistical problems in the analysis of dna sequences..

[47] Rodríguez F, Oliver JL, Marín A, Medina JR (1990). The general stochastic model of nucleotide substitution.. J Theor Biol.

[48] Goldman N, Whelan S (2002). A novel use of equilibrium frequencies in models of sequence evolution.. Mol Biol Evol.

[49] Yang Z (2009). http://abacus.gene.ucl.ac.uk/software/pamlDOC.pdf.

[50] Yang Z (2007). Paml 4: phylogenetic analysis by maximum likelihood.. Mol Biol Evol.

[51] Yang Z (2006). Computational Molecular Evolution.

[52] Whelan S (2007). New approaches to phylogenetic tree search and their application to large numbers of protein alignments.. Syst Biol.

[53] Felsenstein J (1985). Confidence limits on phylogenies: an approach using the bootstrap.. Evolution.

[54] Sugita T, Nakase T (1999). Nonuniversal usage of the leucine cug codon in yeasts: Investigation of basidiomycetous yeast.. J Gen Appl Microbiol.

[55] Santos MA, Tuite MF (1995). The cug codon is decoded in vivo as serine and not leucine in candida albicans.. Nucleic Acids Res.

[56] Kishino H, Hasegawa M (1989). Evaluation of the maximum likelihood estimate of the evolutionary tree topologies from dna sequence data, and the branching order in hominoidea.. J Mol Evol.

[57] Felsenstein J (2004). Inferring Phylogenies.

[58] Akaike H (1974). A new look at the statistical model identification.. Automatic Control, IEEE Transactions on.

[59] Posada D, Buckley TR (2004). Model selection and model averaging in phylogenetics: advantages of akaike information criterion and bayesian approaches over likelihood ratio tests.. Syst Biol.

[60] Burnham KP, Anderson DR (2004). Multimodel Inference: Understanding AIC and BIC in Model Selection.. Sociological Methods Research.

[61] Schwarz G (1978). Estimating the dimension of a model.. The Annals of Statistics.

[62] Weaklim DL (1999). A critique of the bayesian information criterion for model selection.. Sociological Methods Research.

[63] Robinson DF, Foulds LR (1981). Comparison of phylogenetic trees.. Mathematical Biosciences.

[64] Ripplinger J, Sullivan J (2008). Does choice in model selection affect maximum likelihood analysis?. Syst Biol.

[65] Rokas A, Chatzimanolis S (2008). From gene-scale to genome-scale phylogenetics: the data flood in, but the challenges remain.. Methods Mol Biol.

[66] Whelan S, de Bakker PIW, Goldman N (2003). Pandit: a database of protein and associated nucleotide domains with inferred trees.. Bioinformatics.

[67] Scannell DR, Byrne KP, Gordon JL, Wong S, Wolfe KH (2006). Multiple rounds of speciation associated with reciprocal gene loss in polyploid yeasts.. Nature.

[68] Wu Q, James SA, Roberts IN, Moulton V, Huber KT (2008). Exploring contradictory phylogenetic relationships in yeasts.. FEMS Yeast Res.

[69] Nesnidal MP, Helmkampf M, Bruchhaus I, Hausdorf B (2010). Compositional heterogeneity and phylogenomic inference of metazoan relationships.. Mol Biol Evol.

