# Supplementary material for: Addressing Inter-Gene Heterogeneity in Maximum Likelihood Phylogenomic Analysis: Yeasts Revisited
Source: PLoS One. 2011 Aug 5;6(8):e22783. doi: 10.1371/journal.pone.0022783 (PMC3151265; doi:10.1371/journal.pone.0022783)
Supplement: Text S1 — Additional analyses investigating the effects of model choice and between-gene heterogeneity in the single-gene dataset. (PDF) [file pone.0022783.s005.pdf]

Addressing Inter-Gene Heterogeneity in Maximum Likelihood Phylogenomic Analysis: Yeasts Revisited

Jaqueline Hess[1,2*], Nick Goldman[2],
1 Department of Organismic & Evolutionary Biology, Harvard University, Cambridge, MA, USA
2 EMBL-European Bioinformatics Institute, Wellcome Trust Genome Campus, Hinxton, Cambs. CB10 1SD, UK
∗ E-mail: jhess@oeb.harvard.edu

Supporting Text S1

Statistical support for discrepancies between single-gene ML trees reconstructed using different models of evolution

We examined the bootstrap distributions for ML trees reconstructed using different models of evolution to examine the statistical support for the discrepancies between alternative topologies proposed for the same gene. Gene trees were reconstructed using Leaphy with 100 bootstrap replicates each and the JC + Γ, HKY + Γ and REV + Γ models of evolution. Supplementary Figure 1 shows the distributions of shared (red) and variable (blue) nodes in pairwise comparison between models. The support for discrepancies between JC + Γ, the simplest and most unrealistic model used, and the two more complex models is mostly below 70%, there is however a considerable amount of well-supported conflict (Fig. S1A, B). The comparison between HKY + Γ and REV + Γ shows fewer conflicting nodes overall and only very little highly-supported conflict, indicating an improvement in model fit (Fig. S1C). This increase in agreement shows that results improve when better models are used and underlines the importance of finding models that fit the data well.

Variation in single-gene dataset

We explored the heterogeneity in the evolutionary forces acting on the 343 genes in our dataset by investigating the distributions of α, the shape parameter of the gamma distribution used to model across-site rate variation as well as of the transition/transversion ratios (R) estimated for each of those genes (see main text). To see whether the amount of heterogeneity encountered was merely due to noisy estimation of the parameters we examined whether the distance from the mean of their respective distributions for each estimate was associated with alignment length (Fig. S2A left, B for α and R respectively). For α we also considered the distribution of the standard error of each estimate in relation to alignment length to see whether this comparison is appropriate (Fig. S2A right). (Standard error estimates for R were not available.)

The distribution of the standard error of α (Fig. S2A right) shows that there is a clear relationship between gene length and the accuracy of parameter estimation. In contrast, the distribution of the parameter itself (Fig. S2A left), although arguably somewhat associated with gene length, shows numerous estimates close to the population mean for very short alignments and *vice versa*, plenty of estimates on long alignments that are far from the mean. This underlines that the variation of α that we are encountering across the 343 genes is not solely due to accuracy of parameter estimates but is capturing some of the heterogeneity inherent in our data. Similarly, the difference from the mean estimate of R is weakly associated with alignment length but again the shape of the distribution suggests that alignment length alone is not explaining the variation encountered.
