# Supplementary material for: Addressing Inter-Gene Heterogeneity in Maximum Likelihood Phylogenomic Analysis: Yeasts Revisited
Source: PLoS One. 2011 Aug 5;6(8):e22783. doi: 10.1371/journal.pone.0022783 (PMC3151265; doi:10.1371/journal.pone.0022783)
Supplement: Text S2 — BIC model testing. (PDF) [file pone.0022783.s006.pdf]

Addressing Inter-Gene Heterogeneity in Maximum Likelihood Phylogenomic Analysis: Yeasts Revisited

Jaqueline Hess[1,2*], Nick Goldman[2],
1 Department of Organismic & Evolutionary Biology, Harvard University, Cambridge, MA, USA
2 EMBL-European Bioinformatics Institute, Wellcome Trust Genome Campus, Hinxton, Cambs. CB10 1SD, UK
∗ E-mail: jhess@oeb.harvard.edu

Supporting Text S2

BIC model testing

The AIC score is known to favour parameter-rich models under some conditions while the BIC is generally considered to be more conservative [1, 2]. In order to obtain a conservative estimate of model-fit we additionally calculated BIC score [3] for each of our comparisons. As for the AIC_c (see main text), we calculated the penalty term on a per-partition basis. The BIC is then defined as:

$$BIC = -2lnL + \sum_{\text{partitions } i} k_i \log(n_i) \quad (1)$$

where lnL is the maximised log-likelihood of the data, k_i is the number of parameters estimated for partition i with $k = \sum_i k_i$, and n_i is the sample size (number of alignment positions) for partition i with $n = \sum_i n_i$. The results of this are shown in Table S1 and Figure S3.

BIC results for the nucleotide models were very similar to the ones obtained using LRTs or AIC_c (see Table 1 and Figure 4 in the main text). All three tests support the use of complex models, treating each codon position separately. Furthermore, partitioned models were always preferred over concatenated models showing that even a conservative test supports partitioning despite the large number of additional parameters. The BIC, however, selected the REV+Γ+G_4 as the best model as opposed to REV+Γ+G_1 selected by the other two tests. Examination of the AIC_c results in Figure 4 in the main text shows that the gain of information with respect to the number of parameters added between those models is not as great as for other comparisons, suggesting that the model is nearing an optimal level of complexity.

The results for amino acid analysis differed in that the BIC selected concatenated models over partitioned ones. The interpretation of this is somewhat unclear, seeing that the AIC_c is often considered too liberal and the BIC as too conservative, and further study is needed to determine which of the tests applied here is most appropriate for these kind of data. Nevertheless, in this instance the choice of optimal tree is not affected.

References

1. Weaklim DL (1999) A critique of the bayesian information criterion for model selection. Sociological Methods Research 27: 359-397.

2. Burnham KP, Anderson DR (2004) Multimodel Inference: Understanding AIC and BIC in Model Selection. Sociological Methods Research 33: 261-304.

3. Schwarz G (1978) Estimating the dimension of a model. The Annals of Statistics 6: 461-464.

Table 1. BIC test statistics for model tests performed on the supermatrix dataset.

	Concatenated		Partitioned	
**Model**	**ML tree**	**ΔBIC**	**ML tree**	**ΔBIC**
REV	A	886041	A	832326)
REV+Γ	B	327128	B	277374
REV+Γ+G_0	C	179000	C	120364
REV+Γ+G_2	C	175568	C	119900
REV+Γ+G_3	C	58536	C	10374
REV+Γ+G_4	C	40188	C	0
REV+Γ+G_1	C	20364	C	80015
WAG+Γ	C	21951	C	27007
LG+Γ	C	0	C	1612

ML trees and test statistics for model tests performed on the supermatrix dataset. Models used are as in Figure 1 in the main text. ΔBIC is the difference in BIC between a model and the best-fitting model. Trees A and B are shown in Supplementary Figure S4
